# Accessory Subunits of the Matrix Arm of Mitochondrial Complex I with a Focus on Subunit NDUFS4 and Its Role in Complex I Function and Assembly

**DOI:** 10.3390/life11050455

**Published:** 2021-05-19

**Authors:** Flora Kahlhöfer, Max Gansen, Volker Zickermann

**Affiliations:** 1 Structural Bioenergetics Group, Institute of Biochemistry II, Medical School, Goethe University Frankfurt, Max-von-Laue Str. 9, D-60438 Frankfurt, Germany; flora.kahlhoefer@gmail.com (F.K.); s0915628@stud.uni-frankfurt.de (M.G.); 2 Centre for Biomolecular Magnetic Resonance, Institute for Biophysical Chemistry, Goethe University Frankfurt, Max-von-Laue-Str. 9, D-60438 Frankfurt, Germany; 3 Department of Cardiology, Internal Medicine, Goethe University Frankfurt, Theodor-Stern-Kai 7, D-60590 Frankfurt, Germany

**Keywords:** mitochondrial disease, Leigh syndrome, NADH dehydrogenase, respiratory chain, oxidative phosphorylation, assembly factor

## Abstract

NADH:ubiquinone-oxidoreductase (complex I) is the largest membrane protein complex of the respiratory chain. Complex I couples electron transfer to vectorial proton translocation across the inner mitochondrial membrane. The L shaped structure of complex I is divided into a membrane arm and a matrix arm. Fourteen central subunits are conserved throughout species, while some 30 accessory subunits are typically found in eukaryotes. Complex I dysfunction is associated with mutations in the nuclear and mitochondrial genome, resulting in a broad spectrum of neuromuscular and neurodegenerative diseases. Accessory subunit NDUFS4 in the matrix arm is a hot spot for mutations causing Leigh or Leigh-like syndrome. In this review, we focus on accessory subunits of the matrix arm and discuss recent reports on the function of accessory subunit NDUFS4 and its interplay with NDUFS6, NDUFA12, and assembly factor NDUFAF2 in complex I assembly.

## 1. Introduction

Mitochondrial complex I (proton pumping NADH:ubiquinone oxidoreductase) is the largest and most intricate membrane protein complex of the respiratory chain [1,2,3,4]. It is a redox-driven proton pump that couples electron transfer from NADH to ubiquinone (Q) with vectorial proton translocation across the inner mitochondrial membrane. With a proton pump stoichiometry of 4 H^+^ per NADH consumed, complex I contributes about 40% of the proton motive force that drives ATP synthase. Mitochondrial complex I from a broad range of species can reversibly switch from an active A form into an inactive D form [5,6]. The A/D transition is thought to protect against excessive formation of reactive oxygen species [7,8]. The structure of complex I has been determined by X-ray crystallography [9,10] and cryo-EM [1,11,12,13,14,15,16] and is now well described.

**Figure 1:**
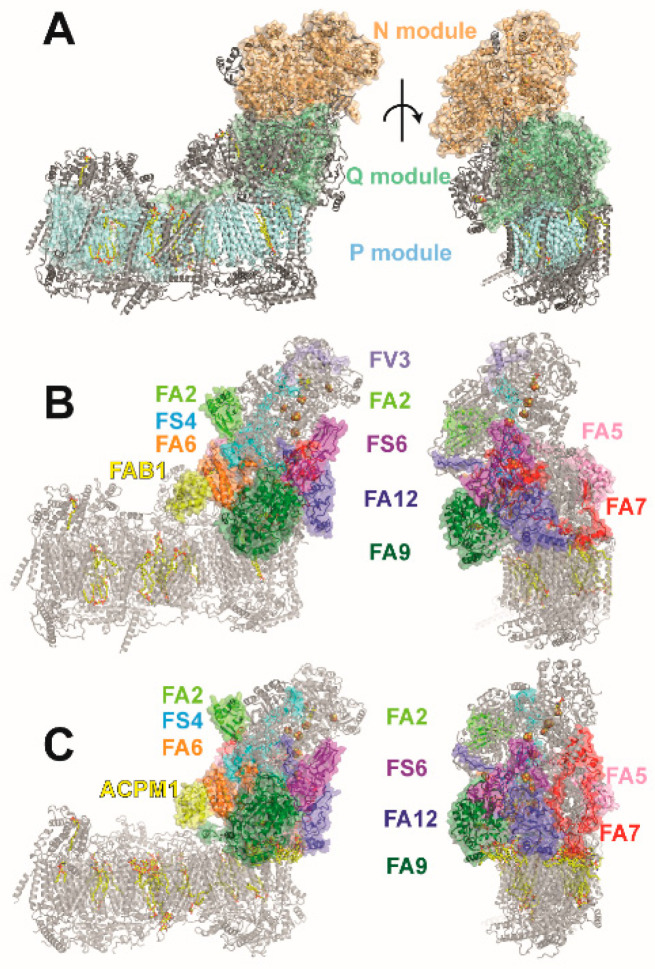
Functional modules of complex I and accessory subunits of the matrix arm. (**A**) The central subunits of complex I are assigned to functional modules for NADH oxidation (N module, orange), ubiquinone reduction (Q module, green), and proton pumping (P module, cyan). (**B**) Accessory subunits of the matrix arm of human complex I (PDB ID: 5xtd) are shown in color, all other subunits are shown in gray. (**C**) Accessory subunits of the matrix arm of *Y. lipolytica* complex I (PDB ID: 6rfr; color code as in (**B**)); the sulfur transferase subunit ST1 is not part of the model; note that FV3 is not present in *Y. lipolytica* complex I.

Mammalian complex I comprises 45 subunits [17]. We have established *Yarrowia lipolytica* as a yeast genetic model organism to study eukaryotic complex I [18]. *Y. lipolytica* complex I comprises 43 subunits of which 40 are orthologues of mammalian complex I [19]. In this review, we use the nomenclature for human complex I also for orthologous proteins from other organisms (Table 1). The large number of polypeptides is divided into central subunits and accessory subunits [20,21]. The 14 central subunits are conserved from bacteria to man and are assigned to three functional modules [21]. The N module (central subunits NDUFS1, NDUFV1, NDUFV2) for NADH oxidation and the Q module (central subunits NDUFS2, NDUFS3, NDUFS7, NDUFS8) for Q reduction are located in the matrix arm of complex I (Figure 1, Table 1). In the membrane arm, the P module (central subunits ND1 to ND6 and ND4L) for proton translocation is subdivided in a proximal P_P_ and a distal P_D_ module [22]. The genes for the seven central subunits of the membrane arm represent a substantial part of the mitochondrial genome. All other complex I subunits and all assembly factors are encoded by nuclear DNA. The N module harbors the NADH oxidation site with the initial electron acceptor FMN. The N and Q modules together comprise eight FeS clusters [23,24]. Cluster N1a is thought to have a function for transient storage of electrons to prevent excessive ROS formation and/or to control NADH binding in the active site [25]. The other seven FeS clusters are arranged in an electron transfer chain connecting the NADH oxidation site with the Q reduction site [26]. Cluster N2 is the immediate electron donor for Q. In contrast to other Q reactive enzymes, the Q reduction site of complex I is buried in the protein structure and is located remotely from the membrane phase [27]. The hydrophobic Q has to transit through a tunnel into the Q module to receive electrons from N2 [9,28,29]. It is generally accepted that the energy driving the proton pumps is released in the Q module. However, the coupling mechanism of complex I has remained controversial.

The large majority of accessory subunits is only found in eukaryotic complex I. A notable exception are subunits NDUFS4, NDUFS6, and NDUFA12 that are already present in complex I from α-proteobacteria [30]. The accessory subunits are arranged around the core of central subunits [10,16,31,32]. In general, their function is less clear, but in many cases, severe complex I assembly defects were found after knock out (KO) of individual genes coding for accessory subunits in human cell lines [33].

Mutations causing a broad spectrum of pathological conditions were reported for central and accessory subunits and assembly factors, and they have been reviewed recently [32,34,35,36].

Here, we focus on accessory subunit NDUFS4 in the matrix arm of complex I and the interplay of accessory subunits with assembly factor NDUFAF2 during the attachment of the N module.

## 2. Accessory Subunits of the Matrix Arm in Yeast and Mammalian Complex I

The matrix arm of mammalian and yeast complex I comprises 10 accessory subunits. Overall, the same set of subunits is found, but subunit NDUFV3 of mammalian complex I is not present in the yeast enzyme complex. NDUFV3 is the only subunit for which tissue specific isoforms have been reported [37,38,39]. On the other hand, only *Y. lipolytica* complex I is associated with the sulfur transferase subunit ST1 [40,41]. Binding of ST1 is substoichiometric and the deletion of the ST1 gene has no impact on complex I function or biogenesis.

NDUFA9 is the largest accessory subunit of the matrix arm. It has the fold of a short chain dehydrogenase [42] and binds NADPH [43]. The cofactor is present in all structures of the eukaryotic complex with sufficient resolution and is therefore a tightly bound component of the subunit [1,11]. The NADPH molecule is too far away from the nearest FeS cluster to allow electron transfer and its function remains unknown. It has been shown recently that NDUFA9 binds the head groups of several phospholipid molecules, which is remarkable for a subunit of the peripheral arm [19]. The subunit is thought to undergo a conformational change in the A/D transition [44] and the relaxation of the protein structure in the C-terminal domain of the subunit has been reported for the D form of mammalian complex I [45].

Mammalian complex I binds two copies of the mitochondrial acyl carrier protein (ACPM) subunit NDUFAB1. In contrast, the yeast enzyme comprises two different but closely related ACPM variants [46]. In all cases, a fatty acid is appended to the phosphopantethein group of the ACPM [47,48]. This fatty acid is inserted into the interior of a mitochondrial LYR (Lys-Tyr-Arg motif) protein that forms a heterodimer with an ACPM [32,49,50]. The mitochondrial LYR proteins were initially implicated in FeS cluster biogenesis [51], but are now recognized to be associated with different macromolecular complexes in the mitochondrion [49]. ACPM/LYRM heterodimers are bound to the Q module of complex I (NDUFAB1α/NDUFA6) [50] and to the tip of the membrane arm (NDUFABβ/NDUFB9) [52]. It is interesting to note that free NDUFAB1 has an essential function in mitochondrial fatty acid synthesis to generate the octanoic acid precursor for lipoic acid [53]. The ACPMs associated with complex I carry longer chain fatty acids and a regulatory function is debated [54]. We have shown that binding of the LYRM protein NDUFA6 to the matrix arm is essential for the Q reductase activity [50]. More recently, we determined the structures of NDUFA6 mutants and showed that single exchanges at the contact site with the functionally important ND3 loop have a strong impact on the interface region of the matrix and membrane arms [55].

NDUFA5 has been noticed in connection with the A/D transition, because the interface of this subunit and accessory subunit NDUFA10 must rearrange during deactivation [45]. Since NDUFA10 is lacking in complex I from *Y. lipolytica*, the longer lifetime of the A form in mammalian complex I might be connected with this specific structural feature [13].

NDUFA2 has a thioredoxin-like fold, but its function has remained unclear. In mammals, the subunit has two cysteine residues, but in *Y. lipoytica*, only one cysteine is conserved.

The three subunits NDUFS4, NDUFS6, and NDUFA12 are distinguished by the fact that they are already found in complex I from α proteobacteria [30]. NDUFS4 has attracted a lot of attention, because it is a hot spot for pathogenic mutations. Knock-out mouse models (*Ndufs4* KO) are widely used to study Leigh syndrome (LS) [56,57]. Moreover, NDUFS4 can be singled out because, in mammalian species, it harbors a canonical serine phosphorylation site [58]. However, analysis of bovine complex I by mass spectrometry did not provide evidence for phosphorylation of the subunit [59,60]. Phosphorylation is thought to play an important role during import and/or maturation of the precursor protein [61,62]. NDUFS6 has a zinc binding site [63]. It is interesting to note that NDUFA12 is a paralog of assembly factor NDUFAF2 [64]. Several lines of evidence have indicated that the interplay of subunits NDUFS4, NDUFS6, and NDUFA12 with assembly factor NDUFAF2 is critical for the attachment of the N module to nascent complex I.

## 3. Leigh Syndrome and the *Ndufs4* KO Mouse Model

In humans, inactivation of the NDUFS4 gene on chromosome 5 is known to cause severe neurologic disorders [65,66,67,68]. In most cases, LS or Leigh-like syndrome is diagnosed (Table 2) [69,70,71,72,73,74,75]. LS is a rare disease with a prevalence of roughly 1:40.000 live births and a generally poor prognosis [76,77,78]. A recent meta-analysis showed that 35% of LS cases are associated with defects in respiratory complex I [79]. In 2016, a ratio of 22 cases of NDUFS4-linked LS for a group of 198 patients with complex I-linked LS was reported [73]. Genotyping of microsatellite DNA markers and array-comparative genomic hybridization has been utilized for diagnosis and might be used for patients with a high pre-test probability in the future [80,81]. Blue native (BN) PAGE consistently revealed abnormal assembly profiles in skin fibroblasts from affected patients and was proposed as a reliable and specific screening method [82]. *Ndufs4* KO mouse models as well as human and murine cell lines have been used extensively to study LS and to explore strategies to counteract the pathophysiological consequences of complex I deficiency [56,57]. Attempts to alleviate disease progression such as expression of plant NDH-2 [83], administration of redox-modulators [84], or targeting of NAD^+^-metabolism [85,86] have been reported. Inhibition of mTOR by rapamycin was shown to dramatically improve survival and health in *Ndufs4* KO mice [87], probably by rescuing a dysfunctional α-ketoglutarate/glutamate/glutamine metabolic axis [88]. The metabolite α-ketoglutarate is thought to sustain sufficient OXPHOS capacity and substrate level phosphorylation even when complex I activity is compromised [89]. In addition, there is evidence that glutamatergic neurons, in particular, drive disease development [88]. The link between mTOR inhibition and the neuron-specific neurotransmitter metabolism opens up a further possible explanation for the positive effect of rapamycin. mTOR is present in two distinct complexes, mTORC1 and mTORC2. mTORC2 was initially described as rapamycin insensitive; however, chronic rapamycin treatment is thought to decrease the formation of new functional mTORC2 [90], resulting in a decrease of PKC-β-dependent pro-inflammatory signaling [91]. Rapamycin treatment thus exerts its positive effect via the inhibition of both mTORC complexes, resulting in changes in metabolism and a decreased tendency to inflammation. In another promising approach at the preclinical stage, it was shown that hypoxia treatment with 11% O_2_ not only ameliorated symptoms but, in fact, led to the reversal of neurological impairment in the *Ndufs4* KO mouse model [92,93,94]. It was recently demonstrated that hypoxic breathing normalizes a detrimental hyperoxia in brain tissue, while activation of the hypoxia-inducible factor (HIF) is not a crucial factor [95]. A new perspective on LS has recently been opened by the observation that switching from glycolytic metabolism to OXPHOS is critical for early neuronal morphogenesis [96]. Defective metabolic reprogramming due to mutations in OXPHOS complexes is thought to be incompatible with normal brain development and might lead to early termination of pregnancy in more cases than previously known.

Gene therapy approaches in the *Ndufs4* KO mouse model were also pursued as an alternative to pharmacological therapy options [111,112]. Adeno-associated viral vector (AAV)-based gene replacement showed promising results in *Ndufs4* KO mice, but differences in the blood brain barrier between mouse and human are still an obstacle for future clinical applications [112].

## 4. NDUFS4-Linked Complex I Dysfunction at the Molecular Level

Several lines of evidence indicate that NDUFS4 plays a role in the late stage of complex I assembly [66,68,113,114]. In animal models and patient cell lines, quantitative mass spectrometry showed that deletion of NDUFS4 caused an increase of assembly factor NDUFAF2 and induced a near complete loss of accessory complex I subunit NDUFA12 [115]. In BN PAGE, an 830 kDa subcomplex harbouring NDUFAF2, but lacking the N module, has been observed. However, in intact tissue, substantial rotenone sensitive Q reductase activity was found, which argues against the complete loss of the N module under in vivo conditions [116]. It is interesting to note that integration of complex I into supercomplexes appears to have a stabilizing function for complex I lacking NDUFS4 [117].

**Figure 2:**
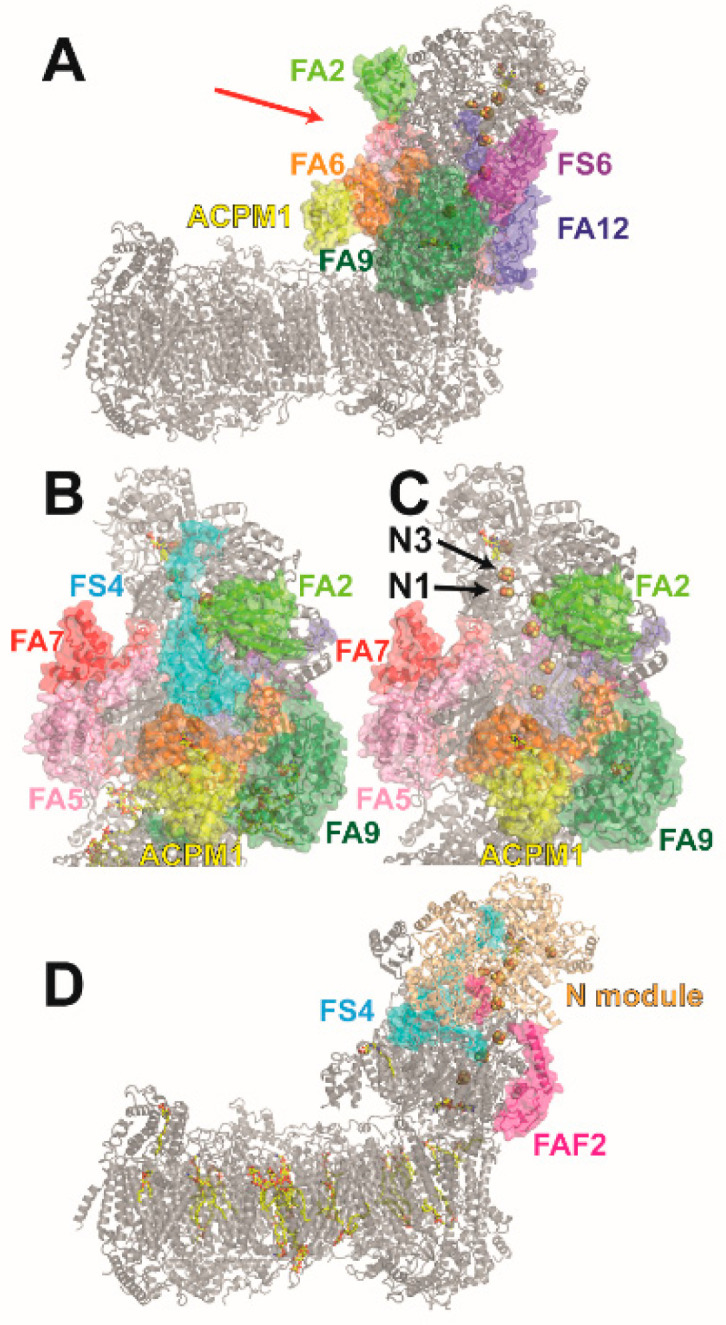
Structure of complex I lacking NDUFS4 and of an assembly intermediate harboring assembly factor NDUFAF2. (**A**) Cryo-EM structure of *Y. lipolytica* complex I purified from *ndufs4*Δ strain (PDB ID: 6rfs); the red arrow indicates direction of view for (**B**) and (**C**). (**B**) Detail view on NDUFS4 in wild type *Y. lipolytica* complex I (PDB ID: 6rfr), direction of view (see (**A**)). (**C**) Same as (**B**) for *ndufs4*Δ mutant (PDB ID: 6rfs); FeS clusters N1b and N3 are solvent exposed. (**D**) Structure of complex I assembly intermediate purified from *Y. lipolytica ndufs6*Δ strain (PDB ID: 6rfq). The assembly intermediate harbors assembly factor NDUFAF2 and all subunits, except NDUFS6 and NDUFA12. For clarity, only NDUFAF2 and NDUFS4 are shown in color. The position of NDUFAF2 matches the position of NDUFA12 in wild type complex I.

We have studied the impact of a NDUFS4 gene deletion on complex I function and assembly in the aerobic yeast *Y. lipolytica* [118]. We found that in the yeast KO strain, complex I levels were decreased and ubiquinone (Q) reductase activity in membranes was reduced. Complexome profiling of intact mitochondria showed that assembly factor NDUFAF2 was bound to complex I, but in clear contrast to the situation observed for mammalian species [115], we did not find a substantial decrease of NDUFA12. In the yeast system, large scale purification of complex I is straightforward. We found that in purified complex I from the NDUFS4 deletion strain, all subunits except NDUFS4 were present and the amount to NDUFAF2 was clearly substoichometric. This suggests that NDUFAF2 was only loosely attached to complex I before solubilization and was easily removed during protein purification. The purified complex showed reduced ubiquinone reductase activity while the formation of ROS under turnover conditions was increased. The EPR spectrum of mutant complex I showed a marked change in the N1b and N3 signals. The cryo-EM structure of the mutant (Figure 2) [19] offered a straightforward explanation for the biochemical and spectroscopic data. We found that the absence of NDUFS4 exposes clusters N1b and N3 to solvent. Thus, the change in EPR spectra is caused by the loss of the shielding function of the accessory subunit. Interestingly, in *T. thermophilus* complex I, the NDUFS1 subunit has an extra loop that partially matches the position of the NDUFS4 subunit in mitochondrial complex I [118]. The increased ROS formation of the mutant might be linked with the greater solvent accessibility of FeS clusters or a longer dwell-time of electrons on FMN, which is known to be critical for the generation of superoxide [119].

## 5. The Role of NDUFS4, NDUFS6, and NDUFA12 in Complex I Assembly

The intricate assembly pathway of mammalian complex I has been studied in detail [120,121,122]. Five submodules are initially formed and then combined in a stepwise process to yield complete complex I. At least 15 assembly factors are known to associate with submodules and play an indispensable role in the assembly process [120]. Assembly factor NDUFAF2 was originally identified as a c-Myc controlled mitochondrial protein (Mimitin) with similarity to complex I subunit NDUFA12 [123]. Whole genome subtraction of fermentative and non-fermentative yeasts gave strong indications that NDUFAF2 is a complex I assembly factor and a null mutation of the associated gene was shown to cause progressive encephalopathy [110]. Analysis of mutants in the fungus *Neurospora crassa* [114] and complementation assays using human mitochondria derived from patients [113] showed that NDUFAF2 function is tightly associated with the attachment of the N module and that NDUFS4, NDUFS6, and NDUFA12 must work together to release the assembly factor in the final step of complex I biogenesis. Since NDUFAF2 and NDUFA12 are paralogs, it had been proposed that both polypeptides occupy the same position in the mature enzyme complex and in the preceding assembly intermediate [64]. We have shown that deletion of the gene encoding NDUFS6 in *Y. lipolytica* caused accumulation of an assembly intermediate that lacked NDUFA12, while NDUFAF2 remained firmly bound [63]. The Q reductase activity of the NDUFS6 KO mutant was reduced to 44%. Mutations in the zinc binding site stalled complex I assembly to varying degrees. Pathogenic mutations in NDUFS6 have been reported (Table 2) and exchange of a cysteine ligand of the metal binding site was shown to cause fatal neonatal lactic acidosis [103]. Taking advantage of straightforward His-tag affinity purification of *Y. lipolytica* complex I, we obtained a preparation of the assembly intermediate of sufficient quality for high-resolution structure determination by cryo-EM (Figure 2) [19]. The structure shows that in the assembly intermediate NDUFAF2 in fact matches the position of NDUFA12 in mature complex I. The NDUFAF2 structure also clashes with the position of NDUFS6. The structure thus offers a straightforward explanation for why NDUFS6 and NDUFA12 are required for the release of NDUFAF2. At first sight, the role of NDUFS4 was less clear, because the subunit appeared to be separated from the assembly factor binding site. Interestingly, no cryo-EM density was observed for a sequence stretch of about 100 amino acids in the C-terminal part of the assembly factor indicating disorder. A finger-like protrusion of NDUFS4 penetrates a narrow cleft between the N and Q modules and comes close to the site where the NDUFAF2 structure is unresolved. We have proposed that in the assembly intermediate NDUFS4 has already pushed out a domain of the assembly factor which becomes flexible after detachment from the complex. The major part of the assembly factor remains bound because NDUFS6 is lacking and the NDUFS6/NDUFA12 tandem cannot be formed for complete removal of NDUFAF2. In the NDUFS4 KO, a weak association of complex I with NDUFAF2 is possible because the protein surface occupied by this accessory subunit in the wild type is still available for the assembly factor in the mutant. The C-terminal end of the assembly factor binds to NDUFS1 and anchors the N module. We propose that before the binding of NDUFS4, the un-modelled sequence stretch of the assembly factor is bound to the assembly intermediate and forms a platform for the docking of the N module. Thus, the C-terminal part of the assembly factor guides the N module to its attachment site, while the N terminal domain is responsible for a stable connection with the nascent complex. These results give a consistent picture for *Y. lipolytica*, but cannot explain why NDUFAF2 remains firmly bound in mammalian NDUFS4 KO cells [115]. We propose that there is no fundamental difference in the N module assembly but that only the relative contribution of NDUFS4, NDUFS6, and NDUFA12 for the detachment of NDUFAF2 is different. In *Y. lipolytica*, NDUFS4 plays a minor part, while, in mammals, the lack of NDUFS4 precludes NDUFAF2 detachment, which in turn blocks the association with NDUFA12. This may also explain a weaker binding of the N module in the mutant complex I.

A recent report showed that the N module is turned over faster than the rest of complex I [124]. The N module is thought to be more exposed to oxidative damage because of superoxide formation at the FMN cofactor [119,125]. Selective exchange of dysfunctional N module is advantageous, because it has a lower energetic cost than *de novo* synthesis of the complete enzyme complex. Interestingly, the three subunits discussed here are among the group of subunits with the highest exchange rate in agreement with their role in the attachment of the N module.

## 6. Conclusions

The central subunits of complex I harbor all bioenergetic core functions. Nevertheless, there is increasing evidence that mutations in accessory complex I subunits can have dramatic consequences and cause fatal disease. The pathophysiology of NDUFS4-linked Leigh syndrome is increasingly well understood. However, therapeutic approaches are still at an experimental stage. Loss of NDUFS4 affects complex I assembly and causes detrimental structural changes in assembled complex I. While animal models and mammalian cell lines are indispensable to study LS and possible therapeutic approaches, the yeast *Y. lipolytica* offers the advantage of straightforward gene manipulation and large-scale purification of complex I variants for biochemical, spectroscopic, and structural analysis of complex I and complex I variants.

## Figures and Tables

**Table 1:** Subunits of the peripheral arm of respiratory complex I.

*Homo sapiens*	*Yarrowia lipolytica*	*Bos taurus*	Comment
central subunits peripheral arm			
NDUFS1	NUAM	75-kDa	2x Fe_4_S_4_; 1x Fe_2_S_2_
NDUFV1	NUBM	51-kDa	FMN; NADH; 1x Fe_4_S_4_
NDUFS2	NUCM	49-kDa	Q-binding
NDUFS3	NUGM	30-kDa	
NDUFV2	NUHM	24-kDa	1x Fe_2_S_2_
NDUFS8	NUIM	TYKY	2x Fe_4_S_4_
NDUFS7	NUKM	PSST	Q-binding; 1x Fe_4_S_4_
accessory subunits peripheral arm			
NDUFA2	NI8M	B8	
NDUFS4	NUYM	18-kDa/AQDQ	
NDUFS6	NUMM	13-kDa	Zn^2+^
NDUFA12	N7BM	B17.2	paralog of assembly factor NDUFAF2
NDUFA7	NUZM	B14.5a	
NDUFA5	NUFM	B13	
NDUFA9	NUEM	39-kDa	NADPH
NDUFA6	NB4M	B14	LYRM6
NDUFAB1	ACPM1	SDAP	ACPM
NDUFV3		9-kDa	
	ST1		sulfur transferase

**Table 2:** Summary of pathogenic mutations in NDUFS4, NDUFS6, NDUFA12, and NDUFAF2.

Subunit	Mutation DNA	Mutation Protein	Disease	Reference
NDUFS4	c.44 G > A	p.Trp15*	Leigh like syndrome	[65]
	c.44 G > A	no complex I assembly	Leigh like syndrome	[68]
	c.99-1 G > A c.462delA	p.Ser34Ilefs*4 p.Lys154Asnfs*34	Leigh syndrome	[97]
	c.221delC	p.Thr74Ilefs*17	Complex I deficiency	[97]
	c.289delG	p.Tyr97*	Leigh like syndrome	[68]
	c.291delG	p.Trp97*	Leigh syndrome	[73]
	c.316 C > T	p.Arg106*	Leigh like syndrome	[98]
	c.340 T > C	p.Trp114Arg	Leigh syndrome	[99]
	c.355 G > C c.462delA	p.Asp119His p.Lys154Asnfs*34	Leigh syndrome	[72]
	c.393dupA	p.Glu132Argfs*15	Leigh syndrome	[70]
	c.462delA	p.Lys154Asnfs*34	Leigh syndrome	[69]
	c.466-470 AAGTC duplication	frameshift, elongation of the carboxyl terminus by 14 residues	Leigh like syndrome	[68,75]
NDUFS6	c.186+2 T > A	splicing abnormality, deletion	Complex I deficiency	[100]
	c.313_315delAAAG	p.104Lys_106Thrfs	Complex I deficiency	[101]
	c.343 C > A c.309 + 5 G > A	p.Cys115Arg	Leigh syndrome	[102]
	c.344 G > A	p.Cys115Tyr	lactic acidemia	[103]
NDUFA12	c.86G > A	p.Arg29Lys	Leigh syndrome	[104]
	c.178C > T	p.Arg60*	Leigh syndrome	[105]
	c.178C > T	p.Arg60*	Mucolipidosis Type II, Leigh syndrome	[106]
	c.224G > A	p.Trp75*	Leigh syndrome	[104]
	c.253G > T	p.Glu85*	Leigh syndrome	[104]
	c.395delA	p.Lys132Argfs*50	Leigh syndrome	[104]
NDUFA2	c.1A > T	p.M1L	hypotonia, nystagmus, ataxia, acute episodes of encephalopathy	[107]
	c.9G > A	p.Trp3*	Leigh syndrome	[108]
	c.103delA	p.Ile35Serfs*	Leigh syndrome	[97]
	c.114C > G	p.Y38*	Leigh syndrome	[109]
	c.182C > T	p.R45*	progressive encephalopathy	[110]
	c.221G > A	p.Trp74*	Leigh syndrome	[97]

## Data Availability

Not applicable.

## References

[1] Parey K., Wirth C., Vonck J., Zickermann V. (2020). Respiratory Complex I—Structure, Mechanism and Evolution. Curr. Opin. Struct. Biol..

[2] Yoga E.G., Angerer H., Parey K., Zickermann V. (2020). Respiratory Complex I—Mechanistic Insights and Advances in Structure Determination. Biochim. et Biophys. Acta (BBA) Bioenerg..

[3] Hirst J. (2013). Mitochondrial Complex I. Annu. Rev. Biochem..

[4] Sazanov L.A. (2015). A Giant Molecular Proton Pump: Structure and Mechanism of Respiratory Complex I. Nat. Rev. Mol. Cell Biol..

[5] Kotlyar A.B., Vinogradov A.D. (1990). Slow Active/Inactive Transition of the Mitochondrial NADH-Ubiquinone Reductase. Biochim. et Biophys. Acta (BBA) Bioenerg..

[6] Maklashina E., Kotlyar A.B., Cecchini G. (2003). Active/De-Active Transition of Respiratory Complex I in Bacteria, Fungi, and Animals. Biochim. et Biophys. Acta (BBA) Bioenerg..

[7] Chouchani E.T., Methner C., Nadtochiy S.M., Logan A., Pell V.R., Ding S., James A.M., Cochemé H.M., Reinhold J., Lilley K.S. (2013). Cardioprotection by S-Nitrosation of a Cysteine Switch on Mitochondrial Complex I. Nat. Med..

[8] Dröse S., Stepanova A., Galkin A. (2016). Ischemic A/D Transition of Mitochondrial Complex I and its Role in ROS Generation. Biochim. et Biophys. Acta (BBA) Bioenerg..

[9] Baradaran R., Berrisford J.M., Minhas G.S., Sazanov L.A. (2013). Crystal Structure of the Entire Respiratory Complex I. Nat. Cell Biol..

[10] Zickermann V., Wirth C., Nasiri H., Siegmund K., Schwalbe H., Hunte C., Brandt U. (2015). Mechanistic Insight from the Crystal Structure of Mitochondrial Complex I. Science.

[11] Agip A.-N.A., Blaza J.N., Fedor J.G., Hirst J. (2019). Mammalian Respiratory Complex I through the Lens of Cryo-EM. Annu. Rev. Biophys..

[12] Kampjut D., Sazanov L.A. (2020). The Coupling Mechanism of Mammalian Respiratory Complex I. Science.

[13] Grba D.N., Hirst J. (2020). Mitochondrial Complex I Structure Reveals Ordered Water Molecules for Catalysis and Proton Translocation. Nat. Struct. Mol. Biol..

[14] Klusch N., Senkler J., Yildiz Ö., Kühlbrandt W., Braun H.-P. (2021). A Ferredoxin Bridge Connects the Two Arms of Plant Mitochondrial Complex I. Plant Cell.

[15] Soufari H., Parrot C., Kuhn L., Waltz F., Hashem Y. (2020). Specific Features and Assembly of the Plant Mitochondrial Complex I Revealed by Cryo-EM. Nat. Commun..

[16] Guo R., Zong S., Wu M., Gu J., Yang M. (2017). Architecture of Human Mitochondrial Respiratory Megacomplex I2III2IV2. Cell.

[17] Carroll J., Fearnley I.M., Skehel J.M., Shannon R.J., Hirst J., Walker J.E. (2006). Bovine Complex I Is a Complex of 45 Different Subunits. J. Biol. Chem..

[18] Kerscher S., Drose S., Zwicker K., Zickermann V., Brandt U. (2002). Yarrowia Lipolytica, a Yeast Genetic System to Study Mito-chondrial Complex I. Biochim. Biophys. Acta.

[19] Parey K., Haapanen O., Sharma V., Köfeler H., Züllig T., Prinz S., Siegmund K., Wittig I., Mills D.J., Vonck J. (2019). High-Resolution cryo-EM Structures of Respiratory Complex I: Mechanism, Assembly, and Disease. Sci. Adv..

[20] Kmita K., Zickermann V. (2013). Accessory Subunits of Mitochondrial Complex I. Biochem. Soc. Trans..

[21] Brandt U. (2006). Energy Converting NADH: Quinone Oxidoreductase (Complex I). Annu. Rev. Biochem..

[22] Hunte C., Zickermann V., Brandt U. (2010). Functional Modules and Structural Basis of Conformational Coupling in Mitochondrial Complex I. Science.

[23] Ohnishi T. (1998). Iron–Sulfur Clusters/Semiquinones in Complex I. Biochim. et Biophys. Acta (BBA) Bioenerg..

[24] Roessler M.M., King M.S., Robinson A.J., Armstrong F.A., Harmer J., Hirst J. (2010). Direct Assignment of EPR Spectra to Structurally Defined Iron-Sulfur Clusters in Complex I by Double Electron–Electron Resonance. Proc. Natl. Acad. Sci. USA.

[25] Schulte M., Frick K., Gnandt E., Jurkovic S., Burschel S., Labatzke R., Aierstock K., Fiegen D., Wohlwend D., Gerhardt S. (2019). A Mechanism to Prevent Production of Reactive Oxygen Species by Escherichia Coli Respiratory Complex I. Nat. Commun..

[26] Sazanov L.A. (2006). Structure of the Hydrophilic Domain of Respiratory Complex I from Thermus Thermophilus. Science.

[27] Zickermann V., Bostina M., Hunte C., Ruiz T., Radermacher M., Brandt U. (2003). Functional Implications from an Unexpected Position of the 49-kDa Subunit of NADH:Ubiquinone Oxidoreductase. J. Biol. Chem..

[28] Warnau J., Sharma V., Gamiz-Hernandez A.P., Di Luca A., Haapanen O., Vattulainen I., Wikström M., Hummer G., Kaila V.R.I. (2018). Redox-Coupled Quinone Dynamics in the Respiratory Complex I. Proc. Natl. Acad. Sci. USA.

[29] Fedor J.G., Jones A.J.Y., Di Luca A., Kaila V.R.I., Hirst J. (2017). Correlating kinetic and structural data on ubiquinone binding and reduction by respiratory complex I. Proc. Natl. Acad. Sci. USA.

[30] Yip C.-Y., Harbour M.E., Jayawardena K., Fearnley I.M., Sazanov L.A. (2011). Evolution of Respiratory Complex I. J. Biol. Chem..

[31] Zhu J., Vinothkumar K.R., Hirst J.Z.J. (2016). Structure of Mammalian Respiratory Complex I. Nat. Cell Biol..

[32] Fiedorczuk K., Letts J.A., Degliesposti G., Kaszuba K., Skehel G.D.M., Sazanov L.A. (2016). Atomic Structure of the Entire Mammalian Mitochondrial Complex I. Nat. Cell Biol..

[33] Stroud D.A., Surgenor E.E., Formosa L.E., Reljic B., Frazier A.E., Dibley M., Osellame L.D., Stait T., Beilharz T.H., Thorburn D.R. (2016). Accessory Subunits are Integral for Assembly and Function of Human Mitochondrial Complex I. Nat. Cell Biol..

[34] Dang Q.-C.L., Phan D.H., Johnson A.N., Pasapuleti M., AlKhaldi H.A., Zhang F., Vik S.B. (2020). Analysis of Human Mutations in the Supernumerary Subunits of Complex I. Life.

[35] Rodenburg R.J. (2016). Mitochondrial Complex I-Linked Disease. Biochim. et Biophys. Acta (BBA) Bioenerg..

[36] Ghezzi D., Zeviani M. (2018). Human Diseases Associated with Defects in Assembly of OXPHOS Complexes. Essays Biochem..

[37] Bridges H.R., Mohammed K., Harbour M.E., Hirst J. (2017). Subunit NDUFV3 is Present in Two Distinct Isoforms in Mammalian Complex I. Biochim. et Biophys. Acta (BBA) Bioenerg..

[38] Dibley M.G., Ryan M.T., Stroud D.A. (2016). A Novel Isoform of the Human Mitochondrial Complex I Subunit NDUFV3. FEBS Lett..

[39] Guerrero-Castillo S., Cabrera-Orefice A., Huynen M.A., Arnold S. (2017). Identification and Evolutionary Analysis of Tissue-Specific Isoforms of Mitochondrial Complex I Subunit NDUFV3. Biochim. et Biophys. Acta (BBA) Bioenerg..

[40] Abdrakhmanova A., Dobrynin K., Zwicker K., Kerscher S., Brandt U. (2005). Functional Sulfurtransferase is Associated with Mitochondrial Complex I fromYarrowia Lipolytica, but is Not Required for Assembly of its Iron-Sulfur Clusters. FEBS Lett..

[41] Parey K., Brandt U., Xie H., Mills D.J., Siegmund K., Vonck J., Kühlbrandt W., Zickermann V. (2018). Cryo-EM Structure of Respiratory Complex I at Work. eLife.

[42] Jörnvall H., Persson B., Krook M., Atrian S., Gonzalez-Duarte R., Jeffery J., Ghosh D. (1995). Short-Chain Dehydrogenases/Reductases (SDR). Biochemistry.

[43] Abdrakhmanova A., Zwicker K., Kerscher S., Zickermann V., Brandt U. (2006). Tight Binding of NADPH to the 39-kDa Subunit of Complex I is Not Required for Catalytic Activity but Stabilizes the Multiprotein Complex. Biochim. et Biophys. Acta (BBA) Bioenerg..

[44] Babot M., Labarbuta P., Birch A., Kee S., Fuszard M., Botting C.H., Wittig I., Heide H., Galkin A. (2014). ND3, ND1 and 39kDa Subunits are more Exposed in the De-Active form of Bovine Mitochondrial Complex I. Biochim. et Biophys. Acta (BBA) Bioenerg..

[45] Agip A.-N.A., Blaza J.N., Bridges H.R., Viscomi C., Rawson S., Muench S.P., Hirst J. (2018). Cryo-EM Structures of Complex I from Mouse Heart Mitochondria in Two Biochemically Defined States. Nat. Struct. Mol. Biol..

[46] Dobrynin K., Abdrakhmanova A., Richers S., Hunte C., Kerscher S., Brandt U. (2010). Characterization of Two Different Acyl Carrier Proteins in Complex I from Yarrowia Lipolytica. Biochim. et Biophys. Acta (BBA) Bioenerg..

[47] Angerer H., Schönborn S., Gorka J., Bahr U., Karas M., Wittig I., Heidler J., Hoffmann J., Morgner N., Zickermann V. (2017). Acyl Modification and Binding of Mitochondrial ACP to Multiprotein Complexes. Biochim. et Biophys. Acta (BBA) Bioenerg..

[48] Carroll J., Fearnley I.M., Shannon R.J., Hirst J., Walker J.E. (2003). Analysis of the Subunit Composition of Complex I from Bovine Heart Mitochondria*S. Mol. Cell. Proteom..

[49] Angerer H. (2015). Eukaryotic LYR Proteins Interact with Mitochondrial Protein Complexes. Biology.

[50] Angerer H., Radermacher M., Mańkowska M., Steger M., Zwicker K., Heide H., Wittig I., Brandt U., Zickermann V. (2014). The LYR Protein Subunit NB4M/NDUFA6 of Mitochondrial Complex I Anchors an Acyl Carrier Protein and is Essential for Catalytic Activity. Proc. Natl. Acad. Sci. USA.

[51] Adam A.C., Bornhövd C., Prokisch H., Neupert W., Hell K. (2006). The Nfs1 Interacting Protein Isd11 has an Essential Role in Fe/S Cluster Biogenesis in Mitochondria. EMBO J..

[52] Zhu J., King M.S., Yu M., Klipcan L., Leslie A.G.W., Hirst J. (2015). Structure of Subcomplex Iβ of Mammalian Respiratory Complex I Leads to New Supernumerary Subunit Assignments. Proc. Natl. Acad. Sci. USA.

[53] Kastaniotis A.J., Autio K.J., Kerätär J.M., Monteuuis G., Mäkelä A.M., Nair R.R., Pietikäinen L.P., Shvetsova A., Chen Z., Hiltunen J.K. (2017). Mitochondrial Fatty Acid Synthesis, Fatty Acids and Mitochondrial Physiology. Biochim. et Biophys. Acta (BBA) Mol. Cell Biol. Lipids.

[54] Nowinski S.M., Van Vranken J.G., Dove K.K., Rutter J. (2018). Impact of Mitochondrial Fatty Acid Synthesis on Mitochondrial Biogenesis. Curr. Biol..

[55] Yoga E.G., Parey K., Djurabekova A., Haapanen O., Siegmund K., Zwicker K., Sharma V., Zickermann V., Angerer H. (2020). Essential Role of Accessory Subunit LYRM6 in the Mechanism of Mitochondrial Complex I. Nat. Commun..

[56] Ingraham C.A., Burwell L.S., Skalska J., Brookes P.S., Howell R.L., Sheu S.-S., Pinkert C.A. (2009). NDUFS4: Creation of a Mouse Model Mimicking a Complex I Disorder. Mitochondrion.

[57] Breuer M.E., Willems P.H., Smeitink J.A., Koopman W.J., Nooteboom M. (2013). Cellular and Animal Models for Mitochondrial Complex I Deficiency: A Focus on the NDUFS4 Subunit. IUBMB Life.

[58] Papa S., Sardanelli A.M., Cocco T., Speranza F., Scacco S.C., Technikova-Dobrova Z. (1996). The Nuclear-Encoded 18 kDa (IP) AQDQ Subunit of Bovine Heart Complex I is Phosphorylated by the Mitochondrial cAMP-Dependent Protein Kinase. FEBS Lett..

[59] De Rasmo D., Palmisano G., Scacco S., Technikova-Dobrova Z., Panelli D., Cocco T.M., Sardanelli A.M., Gnoni A., Micelli L., Trani A. (2010). Phosphorylation Pattern of the NDUFS4 Subunit of Complex I of the Mammalian Respiratory Chain. Mitochondrion.

[60] Chen R., Fearnley I.M., Peak-Chew S.Y., Walker J.E. (2004). The Phosphorylation of Subunits of Complex I from Bovine Heart Mitochondria. J. Biol. Chem..

[61] De Rasmo D., Panelli D., Sardanelli A.M., Papa S. (2008). cAMP-Dependent Protein Kinase Regulates the Mitochondrial Import of the Nuclear Encoded NDUFS4 Subunit of Complex I. Cell. Signal..

[62] Papa S., De Rasmo D., Scacco S., Signorile A., Technikova-Dobrova Z., Palmisano G., Sardanelli A.M., Papa F., Panelli D., Scaringi R. (2008). Mammalian Complex I: A Regulable and Vulnerable Pacemaker in Mitochondrial Respiratory Function. Biochim. et Biophys. Acta (BBA) Bioenerg..

[63] Kmita K., Wirth C., Warnau J., Guerrero-Castillo S., Hunte C., Hummer G., Kaila V.R.I., Zwicker K., Brandt U., Zickermann V. (2015). Accessory NUMM (NDUFS6) Subunit Harbors a Zn-Binding Site and is Essential for Biogenesis of Mitochondrial Complex I. Proc. Natl. Acad. Sci. USA.

[64] Kensche P.R., Duarte I., Huynen A.M. (2012). A Three-Dimensional Topology of Complex I Inferred from Evolutionary Correlations. BMC Struct. Biol..

[65] Petruzzella V., Vergari R., Puzziferri I., Boffoli D., Lamantea E., Zeviani M., Papa S. (2001). A Nonsense Mutation in the NDUFS4 gene Encoding the 18 kDa (AQDQ) Subunit of Complex I Abolishes Assembly and Activity of the Complex in a Patient with Leigh-Like Syndrome. Hum. Mol. Genet..

[66] Petruzzella V., Papa S. (2002). Mutations in Human Nuclear Genes Encoding for Subunits of Mitochondrial Respiratory Complex I: The NDUFS4 Gene. Gene.

[67] Petruzzella V., Panelli D., Torraco A., Stella A., Papa S. (2005). Mutations in the NDUFS4 Gene of Mitochondrial Complex I Alter Stability of the Splice Variants. FEBS Lett..

[68] Scacco S., Petruzzella V., Budde S., Vergari R., Tamborra R., Panelli D., Heuvel L.P.V.D., Smeitink J.A., Papa S. (2003). Pathological Mutations of the Human NDUFS4 Gene of the 18-kDa (AQDQ) Subunit of Complex I Affect the Expression of the Protein and the Assembly and Function of the Complex. J. Biol. Chem..

[69] Anderson S.L., Chung W.K., Frezzo J., Papp J.C., Ekstein J., DiMauro S., Rubin B.Y. (2008). A Novel Mutation in NDUFS4 Causes Leigh Syndrome in an Ashkenazi Jewish Family. J. Inherit. Metab. Dis..

[70] Lamont R.E., Beaulieu C.L., Bernier F.P., Sparkes R., Innes A.M., Jackel-Cram C., Ober C., Parboosingh J.S., Lemire E.G. (2016). A Novel NDUFS4 Frameshift Mutation Causes Leigh Disease in the Hutterite Population. Am. J. Med. Genet. Part A.

[71] Budde S.M.S., Heuvel L.P.W.J.V.D., Smeets R.J.P., Skladal D., Mayr J.A., Boelen C., Petruzzella V., Papa S., Smeitink J.A.M. (2003). Clinical Heterogeneity in Patients with Mutations in the NDUFS4 Gene of Mitochondrial Complex I. J. Inherit. Metab. Dis..

[72] Leshinsky-Silver E., Lebre A.-S., Minai L., Saada A., Steffann J., Cohen S., Rötig A., Munnich A., Lev D., Lerman-Sagie T. (2009). NDUFS4 Mutations cause Leigh Syndrome with Predominant Brainstem Involvement. Mol. Genet. Metab..

[73] Ortigoza-Escobar J.D., Oyarzabal A., Montero R., Artuch R., Jou C., Jiménez C., Gort L., Briones P., Muchart J., López-Gallardo E. (2016). Ndufs4 Related Leigh Syndrome: A Case Report and Review of the Literature. Mitochondrion.

[74] Finsterer J., Zarrouk-Mahjoub S. (2017). NDUFS4-Related Leigh Syndrome in Hutterites. Am. J. Med. Genet. Part A.

[75] Heuvel L.V.D., Ruitenbeek W., Smeets R., Gelman-Kohan Z., Elpeleg O., Loeffen J., Trijbels F., Mariman E., de Bruijn D., Smeitink J. (1998). Demonstration of a New Pathogenic Mutation in Human Complex I Deficiency: A 5-bp Duplication in the Nuclear Gene Encoding the 18-kD (AQDQ) Subunit. Am. J. Hum. Genet..

[76] Rahman S., Blok R.B., Dahl H.-H.M., Danks D.M., Kirby D.M., Chow C.W., Christodoulou J., Thorburn D.R. (1996). Leigh syndrome: Clinical Features and Biochemical and DNA Abnormalities. Ann. Neurol..

[77] Leigh D. (1951). Subacute Necrotizing Encephalomyelopathy in an Infant. J. Neurol. Neurosurg. Psychiatry.

[78] Gerards M., Sallevelt S.C., Smeets H.J. (2016). Leigh Syndrome: Resolving the Clinical and Genetic Heterogeneity Paves the Way for Treatment Options. Mol. Genet. Metab..

[79] Chang X., Wu Y., Zhou J., Meng H., Zhang W., Guo J. (2020). A Meta-Analysis and Systematic Review of Leigh Syndrome: Clinical Manifestations, Respiratory Chain Enzyme Complex Deficiency, and Gene Mutations. Medicine.

[80] Bénit P., Steffann J., Lebon S., Chretien D., Kadhom N., De Lonlay P., Goldenberg A., Dumez Y., Dommergues M., Rustin P. (2003). Genotyping Microsatellite DNA Markers at Putative Disease Loci in Inbred/Multiplex Families with Respiratory Chain Complex I Deficiency Allows Rapid Identification of a Novel Nonsense Mutation (IVS1nt −1) in the NDUFS4 Gene in Leigh Syndrome. Qual. Life Res..

[81] Lombardo B., Ceglia C., Tarsitano M., Pierucci I., Salvatore F., Pastore L. (2014). Identification of a Deletion in the NDUFS4 Gene Using Array-Comparative Genomic Hybridization in a Patient with Suspected Mitochondrial Respiratory Disease. Gene.

[82] Assouline Z., Jambou M., Rio M., Bole-Feysot C., De Lonlay P., Barnerias C., Desguerre I., Bonnemains C., Guillermet C., Steffann J. (2012). A Constant and Similar Assembly Defect of Mitochondrial Respiratory Chain Complex I Allows Rapid Identification of NDUFS4 Mutations in Patients with Leigh Syndrome. Biochim. et Biophys. Acta (BBA) Mol. Basis Dis..

[83] Catania A., Iuso A., Bouchereau J., Kremer L.S., Paviolo M., Terrile C., Bénit P., Rasmusson A.G., Schwarzmayr T., Tiranti V. (2019). Arabidopsis Thaliana Alternative Dehydrogenases: A Potential Therapy for Mitochondrial Complex I Deficiency? Perspectives and Pitfalls. Orphanet J. Rare Dis..

[84] De Haas R., Das D., Garanto A., Renkema H.G., Greupink R., Broek P.V.D., Pertijs J., Collin R.W.J., Willems P., Beyrath J. (2017). Therapeutic Effects of the Mitochondrial ROS-Redox Modulator KH176 in a Mammalian Model of Leigh Disease. Sci. Rep..

[85] Karamanlidis G., Lee C.F., Garcia-Menendez L., Kolwicz S.C., Suthammarak W., Gong G., Sedensky M.M., Morgan P.G., Wang W., Tian R. (2013). Mitochondrial Complex I Deficiency Increases Protein Acetylation and Accelerates Heart Failure. Cell Metab..

[86] Lee C.F., Caudal A., Abell L., Gowda G.A.N., Tian R. (2019). Targeting NAD+ Metabolism as Interventions for Mitochondrial Disease. Sci. Rep..

[87] Johnson S.C., Yanos M.E., Kayser E.-B., Quintana A., Sangesland M., Castanza A., Uhde L., Hui J., Wall V.Z., Gagnidze A. (2013). mTOR Inhibition Alleviates Mitochondrial Disease in a Mouse Model of Leigh Syndrome. Science.

[88] Johnson S.C., Kayser E.-B., Bornstein R., Stokes J., Bitto A., Park K.Y., Pan A., Sun G., Raftery D., Kaeberlein M. (2020). Regional Metabolic Signatures in the Ndufs4(KO) Mouse Brain Implicate Defective Glutamate/α-Ketoglutarate Metabolism in Mitochondrial Disease. Mol. Genet. Metab..

[89] Kayser E.-B., Sedensky M.M., Morgan P.G. (2016). Region-Specific Defects of Respiratory Capacities in the Ndufs4(KO) Mouse Brain. PLoS ONE.

[90] Sarbassov D.D., Ali S.M., Sengupta S., Sheen J.-H., Hsu P.P., Bagley A.F., Markhard A.L., Sabatini D.M. (2006). Prolonged Rapamycin Treatment Inhibits mTORC2 Assembly and Akt/PKB. Mol. Cell.

[91] Martin-Perez M., Grillo A.S., Ito T.K., Valente A.S., Han J., Entwisle S.W., Huang H.Z., Kim D., Yajima M., Kaeberlein M. (2020). PKC Downregulation upon Rapamycin Treatment Attenuates Mitochondrial Disease. Nat. Metab..

[92] Ferrari M., Jain I.H., Goldberger O., Rezoagli E., Thoonen R., Cheng K.-H., Sosnovik D.E., Scherrer-Crosbie M., Mootha V.K., Zapol W.M. (2017). Hypoxia Treatment Reverses Neurodegenerative Disease in a Mouse Model of Leigh Syndrome. Proc. Natl. Acad. Sci. USA.

[93] Jain I.H., Zazzeron L., Goli R., Alexa K., Schatzman-Bone S., Dhillon H., Goldberger O., Peng J., Shalem O., Sanjana N.E. (2016). Hypoxia as a Therapy for Mitochondrial Disease. Science.

[94] Grange R.M., Sharma R., Shah H., Reinstadler B., Goldberger O., Cooper M.K., Nakagawa A., Miyazaki Y., Hindle A.G., Batten A.J. (2021). Hypoxia Ameliorates Brain Hyperoxia and NAD+ Deficiency in a Murine Model of Leigh Syndrome. Mol. Genet. Metab..

[95] Jain I.H., Zazzeron L., Goldberger O., Marutani E., Wojtkiewicz G.R., Ast T., Wang H., Schleifer G., Stepanova A., Brepoels K. (2019). Leigh Syndrome Mouse Model Can Be Rescued by Interventions that Normalize Brain Hyperoxia, but Not HIF Activation. Cell Metab..

[96] Inak G., Rybak-Wolf A., Lisowski P., Pentimalli T.M., Jüttner R., Glažar P., Uppal K., Bottani E., Brunetti D., Secker C. (2021). Defective Metabolic Programming Impairs Early Neuronal Morphogenesis in Neural Cultures and an Organoid Model of Leigh Syndrome. Nat. Commun..

[97] Calvo E.S., Tucker E.J., Compton A., Kirby D.M., Crawford G., Burtt N.P., Rivas M., Guiducci C., Bruno D.L., Goldberger A.O. (2010). High-Throughput, Pooled Sequencing Identifies Mutations in NUBPL and FOXRED1 in Human Complex I Deficiency. Nat. Genet..

[98] Budde S., Heuvel L.V.D., Janssen A., Smeets R., Buskens C., DeMeirleir L., Van Coster R., Baethmann M., Voit T., Trijbels J. (2000). Combined Enzymatic Complex I and III Deficiency Associated with Mutations in the Nuclear Encoded NDUFS4 Gene. Biochem. Biophys. Res. Commun..

[99] Kohda M., Tokuzawa Y., Kishita Y., Nyuzuki H., Moriyama Y., Mizuno Y., Hirata T., Yatsuka Y., Yamashita-Sugahara Y., Nakachi Y. (2016). A Comprehensive Genomic Analysis Reveals the Genetic Landscape of Mitochondrial Respiratory Chain Complex Deficiencies. PLoS Genet..

[100] Kirby D.M., McFarland R., Ohtake A., Dunning C., Ryan M.T., Wilson C., Ketteridge D., Turnbull D.M., Thorburn D.R., Taylor R.W. (2004). Mutations of the Mitochondrial ND1 Gene as a Cause of MELAS. J. Med. Genet..

[101] Pronicka E., Piekutowska-Abramczuk D., Ciara E., Trubicka J., Rokicki D., Karkucińska-Więckowska A., Pajdowska M., Jurkiewicz E., Halat P., Kosińska J. (2016). New Perspective in Diagnostics of Mitochondrial Disorders: Two Years’ Experience with Whole-Exome Sequencing at a National Paediatric Centre. J. Transl. Med..

[102] Ogawa E., Shimura M., Fushimi T., Tajika M., Ichimoto K., Matsunaga A., Tsuruoka T., Ishige M., Fuchigami T., Yamazaki T. (2017). Clinical Validity of Biochemical and Molecular Analysis in Diagnosing Leigh Syndrome: A Study of 106 Japanese Patients. J. Inherit. Metab. Dis..

[103] Spiegel R., Shaag A., Mandel H., Reich D.S., Penyakov M., Hujeirat Y., Saada A., Elpeleg O., Shalev A.S. (2009). Mutated NDUFS6 is the Cause of Fatal Neonatal Lactic Acidemia in Caucasus Jews. Eur. J. Hum. Genet..

[104] Torraco A., Nasca A., Verrigni D., Pennisi A., Zaki M.S., Olivieri G., Assouline Z., Martinelli D., Maroofian R., Rizza T. (2021). Novel NDUFA12 Variants are Associated with Isolated Complex I Defect and Variable Clinical Manifestation. Hum. Mutat..

[105] Ostergaard E., Rodenburg R.J., Brand M.V.D., Thomsen L.L., Duno M., Batbayli M., Wibrand F., Nijtmans L. (2011). Respiratory Chain Complex I Deficiency due to NDUFA12 Mutations as a New Cause of Leigh Syndrome. J. Med. Genet..

[106] Speer R.R., Ezeanya U.C., Beaudoin S.J., Glass K.M., Oji-Mmuo C.N. (2019). Term Neonate Presenting with the Combined Occurrence of Mucolipidosis Type II and Leigh Syndrome. J. Pediatr. Genet..

[107] Barghuti F., Elian K., Gomori J.M., Shaag A., Edvardson S., Saada A., Elpeleg O. (2008). The Unique Neuroradiology of Complex I Deficiency Due to NDUFA12L Defect. Mol. Genet. Metab..

[108] Herzer M., Koch J., Prokisch H., Rodenburg R., Rauscher C., Radauer W., Forstner R., Pilz P., Rolinski B., Freisinger P. (2010). Leigh Disease with Brainstem Involvement in Complex I Deficiency due to Assembly Factor NDUFAF2 Defect. Neuropediatrics.

[109] Hoefs S.J., Dieteren C.E., Rodenburg R.J., Naess K., Bruhn H., Wibom R., Wagena E., Willems P.H., Smeitink J.A., Nijtmans L.G. (2009). Baculovirus Complementation Restores a Novel NDUFAF2 Mutation Causing Complex I Deficiency. Hum. Mutat..

[110] Ogilvie I., Kennaway N.G., Shoubridge E.A. (2005). A Molecular Chaperone for Mitochondrial Complex I Assembly is Mutated in a Progressive Encephalopathy. J. Clin. Investig..

[111] Reynaud-Dulaurier R., Benegiamo G., Marrocco E., Al-Tannir R., Surace E.M., Auwerx J., Decressac M. (2020). Gene Replacement Therapy Provides Benefit in an Adult Mouse Model of Leigh Syndrome. Brain.

[112] Silva-Pinheiro P., Cerutti R., Luna-Sanchez M., Zeviani M., Viscomi C. (2020). A Single Intravenous Injection of AAV-PHP.B-hNDUFS4 Ameliorates the Phenotype of Ndufs4 Mice. Mol. Ther. Methods Clin. Dev..

[113] Lazarou M., McKenzie M., Ohtake A., Thorburn D.R., Ryan M.T. (2007). Analysis of the Assembly Profiles for Mitochondrial- and Nuclear-DNA-Encoded Subunits into Complex I. Mol. Cell. Biol..

[114] Pereira B., Videira A., Duarte M. (2013). Novel Insights into the Role of Neurospora crassa NDUFAF2, an Evolutionarily Conserved Mitochondrial Complex I Assembly Factor. Mol. Cell. Biol..

[115] Adjobo-Hermans M.J., de Haas R., Willems P.H., Wojtala A., Vries S.E.V.E.-D., Wagenaars J.A., Brand M.V.D., Rodenburg R.J., Smeitink J.A., Nijtmans L.G. (2020). NDUFS4 Deletion Triggers Loss of NDUFA12 in Ndufs4 Mice and Leigh Syndrome Patients: A Stabilizing Role for NDUFAF2. Biochim. et Biophys. Acta (BBA) Bioenerg..

[116] Kruse S.E., Watt W.C., Marcinek D.J., Kapur R.P., Schenkman K.A., Palmiter R.D. (2008). Mice with Mitochondrial Complex I Deficiency Develop a Fatal Encephalomyopathy. Cell Metab..

[117] Calvaruso M.A., Willems P., Brand M.V.D., Valsecchi F., Kruse S., Palmiter R., Smeitink J., Nijtmans L. (2011). Mitochondrial Complex III Stabilizes Complex I in the Absence of NDUFS4 to Provide Partial Activity. Hum. Mol. Genet..

[118] Kahlhöfer F., Kmita K., Wittig I., Zwicker K., Zickermann V. (2017). Accessory Subunit NUYM (NDUFS4) is Required for Stability of the Electron Input Module and Activity of Mitochondrial Complex I. Biochim. et Biophys. Acta (BBA) Bioenerg..

[119] Galkin A., Brandt U. (2005). Superoxide Radical Formation by Pure Complex I (NADH:Ubiquinone Oxidoreductase) from Yarrowia lipolytica. J. Biol. Chem..

[120] Formosa L.E., Dibley M., Stroud D.A., Ryan M.T. (2018). Building a complex complex: Assembly of Mitochondrial Respiratory Chain Complex I. Semin. Cell Dev. Biol..

[121] Formosa L.E., Muellner-Wong L., Reljic B., Sharpe A.J., Jackson T.D., Beilharz T.H., Stojanovski D., Lazarou M., Stroud D.A., Ryan M.T. (2020). Dissecting the Roles of Mitochondrial Complex I Intermediate Assembly Complex Factors in the Biogenesis of Complex I. Cell Rep..

[122] Guerrero-Castillo S., Baertling F., Kownatzki D., Wessels H.J., Arnold S., Brandt U., Nijtmans L. (2017). The Assembly Pathway of Mitochondrial Respiratory Chain Complex I. Cell Metab..

[123] Tsuneoka M., Teye K., Arima N., Soejima M., Otera H., Ohashi K., Koga Y., Fujita H., Shirouzu K., Kimura H. (2005). A Novel Myc-Target Gene, mimitin, That Is Involved in Cell Proliferation of Esophageal Squamous Cell Carcinoma*. J. Biol. Chem..

[124] Stepanova A., Sosunov S., Niatsetskaya Z., Konrad C., Starkov A.A., Manfredi G., Wittig I., Ten V., Galkin A., Sosunov S. (2019). Redox-Dependent Loss of Flavin by Mitochondrial Complex I in Brain Ischemia/Reperfusion Injury. Antioxid. Redox Signal..

[125] Kussmaul L., Hirst J. (2006). The Mechanism of Superoxide Production by NADH:Ubiquinone Oxidoreductase (complex I) from Bovine Heart Mitochondria. Proc. Natl. Acad. Sci. USA.

